# Alzheimer's disease phenotype based upon the carrier status of the apolipoprotein E ɛ4 allele

**DOI:** 10.1111/bpa.13208

**Published:** 2023-08-30

**Authors:** Xiao‐Yu Ji, Xin‐Yuan Peng, Hai‐Liang Tang, Hui Pan, Wei‐Tang Wang, Jie Wu, Jian Chen, Nai‐Li Wei

**Affiliations:** ^1^ Department of Neurosurgery The First Affiliated Hospital of Shantou University Medical College Guangdong China; ^2^ Brain Function and Disease Laboratory Shantou University Medical College Guangdong China; ^3^ Fudan University Huashan Hospital, Department of Neurosurgery, State Key Laboratory for Medical Neurobiology Institutes of Brain Science, Shanghai Medical College‐Fudan University Shanghai China; ^4^ Shantou Longhu People's Hospital Shantou Guangdong China

**Keywords:** Alzheimer's disease, apolipoprotein E, lipid metabolism, phenotype

## Abstract

The apolipoprotein E *ɛ4* allele (*APOE4*) is universally acknowledged as the most potent genetic risk factor for Alzheimer's disease (AD). *APOE4* promotes the initiation and progression of AD. Although the underlying mechanisms are unclearly understood, differences in lipid‐bound affinity among the three APOE isoforms may constitute the basis. The protein APOE4 isoform has a high affinity with triglycerides and cholesterol. A distinction in lipid metabolism extensively impacts neurons, microglia, and astrocytes. *APOE4* carriers exhibit phenotypic differences from non‐carriers in clinical examinations and respond differently to multiple treatments. Therefore, we hypothesized that phenotypic classification of AD patients according to the status of APOE4 carrier will help specify research and promote its use in diagnosing and treating AD. Recent reviews have mainly evaluated the differences between *APOE4* allele carriers and non‐carriers from gene to protein structures, clinical features, neuroimaging, pathology, the neural network, and the response to various treatments, and have provided the feasibility of phenotypic group classification based on *APOE4* carrier status. This review will facilitate the application of *APOE* phenomics concept in clinical practice and promote further medical research on AD.

## Graphical abstract

Genetic variation in the APOE genotype form the basis of the clinical phenotype.

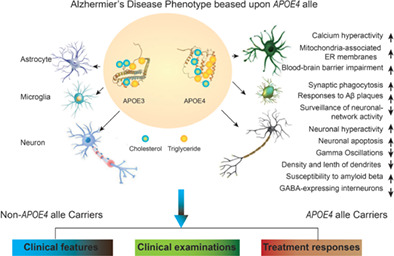

## 1 INTRODUCTION

Alzheimer's disease (AD) is a common neurodegenerative disease in elder people with progressive cognitive decline and psychobehavioral abnormalities [1]. It is also the primary type of dementia, occupying 60%–80% of all cases [2]. It affects 6.5 million Americans aged 65 and older today. By 2060, this number could reach 13.8 million without medical breakthroughs in preventing, curing, or slowing AD [2]. Despite advancements in clinical examination, multi‐omics, and experimental techniques, the exact pathogenic mechanisms are not fully understood. Both epidemiological research and genetic studies have proved that the apolipoprotein E *ɛ4* allele (*APOE4*) exhibited the most potent genetic risk factor for AD with a dose‐dependent manner [3]. Apolipoprotein E (APOE) is the principal triglyceride and cholesterol (CL) carrier and plays a primary role in lipid metabolism and homeostasis in the blood and central nervous system (CNS) [4]. It has three protein isoforms (APO‐E2, E3, and E4) encoded by corresponding allelic variants (*APOE*‐*ɛ2*, *ɛ3*, and *ɛ4*) [5]. Compared to the most frequent allele *APOE3*, the carrier status of *APOE4* can greatly increase the risk of AD by 3–4 times in heterozygotes and 15 times in homozygotes, while *APOE ɛ2* can reduce the risk by 40% [6, 7]. Furthermore, *APOE4* drives pathogenesis involving both amyloid plaques (Aβ) and abnormal phosphorylation of Tau proteins [8]. Considering its detrimental effects, therapeutic approaches based on *APOE4* status are advocated [3].

Significant and extensive differences exist among APOE isoforms carrying status in clinical features, including neuroimaging, pathogenesis, inflammation, and lipid metabolism. Studies on the impact of APOE genotype on AD phenome have made great progress. Integrating the specific role of *APOE4* in AD development, this review summarizes recent progress and discusses the feasibility of phenotypic group classification using *APOE4* carrier status.

## 2 STRUCTURE AND FUNCTION

### 2.1 Synthesis and degradation of APOE

APOE can be synthesized in various organs, including the liver, brain, spleen, and kidneys. The brain possesses the second most APOE production in the whole body [9]. Various cells in the brain can secrete APOE, which are mainly astrocytes [10]. Neurons in the hippocampus and cortex can also synthesize APOE under injury or stress [11].

**FIGURE 1:**
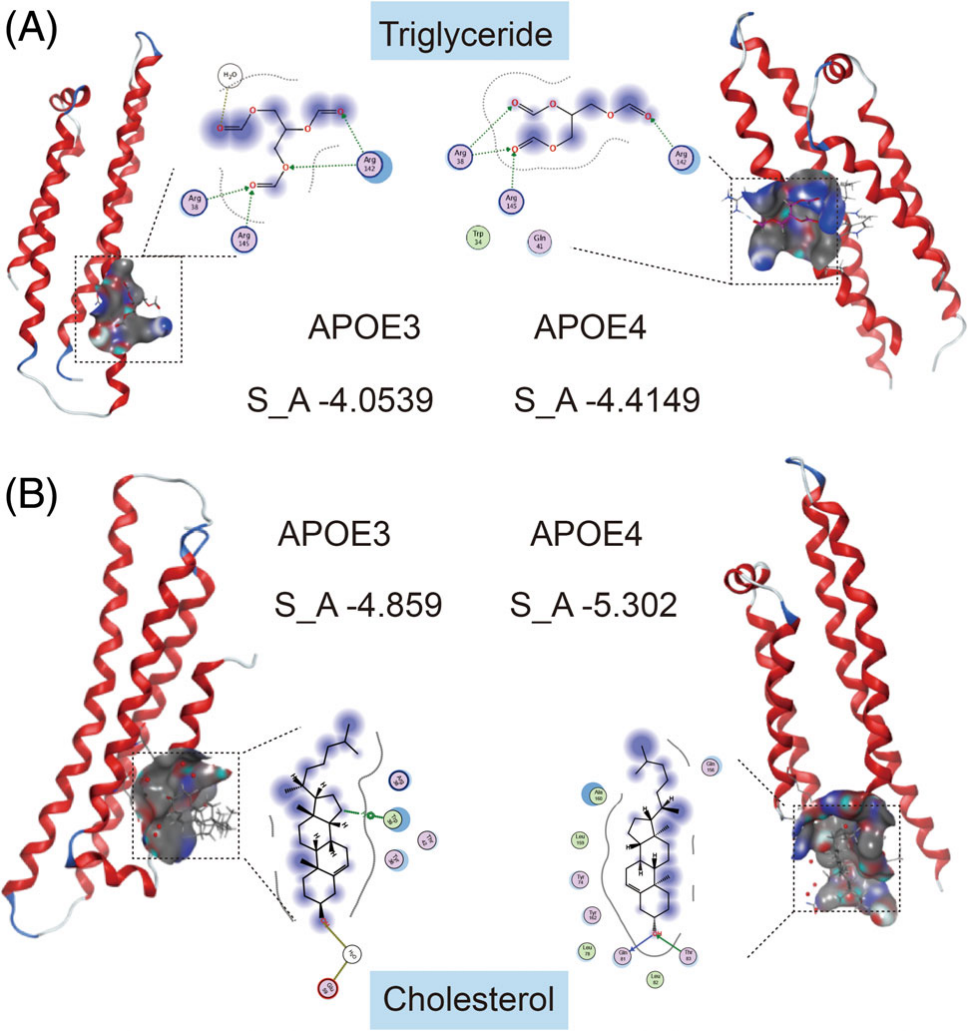
Molecular docking results indicate that the binding affinity of APOE4 with triglycerides (A) and cholesterol (B) is higher than that of APOE3.

The molecular structures and functions of APOE isoforms vary because of different *APOE* genotypes. The protein APOE consists of 299 amino acids (aa), of which N‐terminal domain (1–167 aa) and C‐terminal domain (206–299 aa) are connected by hinges (Figure 1) [5]. The two domains are responsible for the function of APOE, with 135–150 aa being the low‐density lipoprotein receptor (LDLR)‐binding domain and 244–272 aa being the lipid‐binding domain (Figure 1) [5]. The structures of the two domains lead to functional differences among the three isoforms. In APOE3, Cys‐112 approaches Glu‐109 in helix bundle 3 and Arg‐61 in helix bundle 2, affecting ionic bonding in the two helix bundles [12]. Compared to APOE3, Cys‐112 is replaced by Arg‐114 in APOE4, which destroys the ionic effect between Glu‐109 and Arg‐61. The side chain of ARG‐61 is removed from the helical bundle, and a salt bridge is formed between Arg‐61 and Glu‐255, thus forming an interaction between the domains and lowering the ability of APOE4 to bind to lipids (Figure 1) [5, 12]. In addition, a salt bridge exists between Asp‐154 and Arg‐158 in APOE3 and APOE4. In contrast to APOE3 and APOE4, with Arg‐158 substituted by Cys‐158, a salt bridge is formed between Asp‐154 and Arg‐150 in APOE2, which may separate the side chain of Arg‐150 from LDLR binding domain, influencing the ability of APOE2 to bind to LDLR [5, 12]. Meanwhile, mutagenesis of aa could also affect the stability of the protein (APOE4 < APOE3 < APOE2) [13].

Apolipoprotein E undergoes proteolytic cleavage in response to many enzymes in CNS, generating truncated fragments that can be associated with neurofibrillary tangles (NFT) and mitochondrial damage [14]. The fragmentation pattern varied between isoforms, indicating different degrees of damage caused by fragmentation. Therefore, APOE4 is more likely to degenerate than APOE3, and 25 kDa N‐terminal fragment in APOE4 is reduced by half compared to APOE3 brains [15]. However, a discrepancy also exists between the fragments generated by APOE3 and APOE4 [15, 16, 17]. APOE 22 kDa in the N‐terminal domain can bind with their cell surface receptors and induce calcium influx and neurotoxicity. The 22 kDa fragment produced by APOE4 displays higher toxicity than APOE3 [18, 19]. Meanwhile, fragment 1–271 aa in APOE can induce NFT formation in neuronal cells in a manner of APOE4 greater than APOE3 [20, 21]. Furthermore, APOE4 fragment 1–271 aa causes AD‐like Tau pathology and behavioral deficits [17], while fragment 1–272 aa promotes mitochondria dysfunction [21]. Fragments 1–185 can induce IL‐1β and decrease IL‐10 expression, ultimately causing an MMP9/TIMP1 imbalance [22]. In addition, increased intracellular accumulation of Aβ42, which generates reactive oxygen species (ROS) by fragment 1–165 aa, can only be found in APOE4 but not APOE3 [22, 23]. Similarly, only fragments 1–151 aa generated by APOE4 can be trafficked to the nucleus to increase cell death [24]. In brief, proteolytic APOE4 fragments may cause Tau protein phosphorylation, neurofibrillary tangles, cytoskeletal changes, and impairment of mitochondrial function, which may explain why *APOE4* has a higher risk of AD pathogenicity than *APOE3*.

### 2.2 APOE and cholesterol metabolism in the brain

Cholesterol (CL) is a fundamental necessary substance for developing synapses and plays a significant role in synaptogenesis and synaptic stability [25]. CL overload often occurs in adult neurons (Table 1) because they primarily rely on exogenous CL from astrocytes [26]. In addition, CNS has a CL redistribution pathway mediated by APOE because of the presence of a blood‐brain barrier [27]. Briefly, APOE is an important regulatory factor of CNS‐CL metabolism.

**TABLE 1:** Clinical features between *APOE4* carriers and non‐carriers.

Study (years)	Nubmers of patients (*n* = *APOE4* carriers; % = *APOE4* carriers of total patients)	To investigate the impact of *APOE* genotype on clinical features	Findings
*Cognitive behavior*			
D. X. Rasmusson, et al. (1996)	157 AD patients (*n* = 112; 71.3%)	Verbal deficits (MMSE, BNT)	*APOE4* allele was related to the impairment of global cognition but not language [163].
M. Lehtovirta, et al. (1996)	58 AD patients (*n* = 37; 63.8%) and 16 control elder people (*n* = 3, 18.75%).	Neuropsychological characteristics (MMSE, Webster score, Hamilton score, Brief Cognitive ranking score)	AD patients with two ɛ4 alleles are characterized by more severe memory loss and earlier age of onset than those without the ɛ4 allele [164].
M. J. Finton, et al. (2003)	200 AD patients (*n* = 104; 52%)	Cognitive asymmetries (nonverbal ability and verbal cognitive ability)	*APOE4* allele carriers have relatively worse nonverbal as compared to verbal cognitive ability [165].
W. S. Houston, et al. (2005)	52 healthy older participants (*n* = 24; 46.15%)	Cognitive asymmetries (nonverbal ability and verbal cognitive ability)	*APOEɛ4* allele carriers demonstrated a greater frequency of cognitive asymmetric profile than non‐carriers [166].
B. D. Hoyt, et al. (2005)	151 AD patients (*n* = 104; 68.87%)	Global and specific measures of cognitive and functional abilities (MMSE, ADAS, IADL)	*APOE*ɛ4 homozygote is associated with global cognitive functioning and functional abilities [167].
X. Wang, et al. (2015)	42 AD patients (*n* = 16; 38.09%)	Role of *APOE4* in cognitive profiles in AD (COMT, MMSE, CASI C‐2. CERAD)	*APOEε4*+ exhibited poorer performance on recognition performance, but performed better on the late item generation of the verbal fluency task [168].
U. Saeed, et al. (2018)	250 AD patients and 48 DLB patients	Learning, and memory (MMSE, DRS, CVLT )	APOEε4+ performed worse on long‐delay free word recall [169].
*Neuropsychology behaviors*			
C. G. Lyketsos, et al. (1999)	158 AD patients (*n* = 108; 68.35%) and 73 control elder people (*n* = 3, 27.40%)	Depression, delusions, and hallucinations in AD (BRDS, MMSE, past medical history, clinical evaluations)	Prevalence of the various psychiatric disturbances did not differ significantly in AD patients with different *APOE* genotypes [170].
C. Holmes, et al. (1998)	103 depressed AD patients (*n* = 30, 33%), 107 non‐depressed AD patients (*n* = 30, 28%) and 74 elder depression patients (*n* = 9, 12%)	Depressive symptomatology (DSM‐IV criteria, SADS‐L, TICS)	The presence of the *APOE* epsilon 2 allele delayed the process of depressive illness [171].
N. Scarmeas, et al. (2002)	87 AD patients (*n* = 48; 55.17%)	Psychiatric symptomatology incident (CUSPAD)	*APOE4* heterozygote carried a 2.5‐fold risk, whereas the homozygote carried a 5.6‐fold risk for development of delusions [172].
M. W. Bondi, et al. (2003)	81 AD patients (*n* = 44; 54.32%) and 79 control (*n* = 29, 36.71%)	Neuropsychological deficits associated with age: (1) Language: Boston Naming Test, Letter and Category Fluency, and WAIS–R vocabulary; (2) executive functions: modified WCST categories and perseverative errors, trailmaking test Part B; (3) Visuoconstructive and psychomotor skills: WISC–R Block design, WAIS–R digit symbol, trailmaking test part A; (4) Immediate recall: CVLT Trials 1–5 total recall, WMS–R logical memory immediate recall; (5) Delayed recall: CVLT long‐delay freeand cued‐recall, WMS–R logical memory delayed recall, and (6) Recall savings: CVLT percent long‐delay savings, WMS–R percent delayed recall savings.	Age related neuropsychological deficits with the disproportionate saliency of episodic memory and executive function deficits [173].
J. B. Chang, et al. (2004)	135 AD patients (*n* = 59; 43.70%)	Incidental hallucinations and delusions (CASI, CDR, SCID)	The presence of the *APOE*ɛ4 allele carried a 19.0‐fold risk for developing hallucinations and a 3.4‐fold risk for delusions [174].
*Neuroimage*			
M. Lehtovirta, et al. (1996)	58 AD patients (*n* = 59; 63.79%) and 34 control subjects	Relation between SPECT and MRI and apolipoprotein ɛ4 allele in AD	*APOE*ɛ4 homozygous seem to have severe damage in the medial temporal lobe structures early in the disease process and differ from the AD patients with one or no epsilon 4 alleles [175].
M. Yasuda, et al. (1998)	178 AD patients (*n* = 112; 65.17%)	Relationship between the *APOE* epsilon 4 allele and whole brain atrophy (MRI)	*APOE*ɛ4 alleles aggravated brain atrophy [176].
A. Fleisher, et al. (2005)	193 MCI patients (*n* = 112; 65.17%)	Association of sex, *APOE*ɛ4 status, and hippocampal volume in MCI (MRI)	The *APOE*ɛ4 genotype status appears to have a greater deleterious effect on gross hippocampal pathology and memory performance in women than in men [177].
L. A. van de Pol, et al. (2007)	323 MCI patients	Hippocampal atrophy rate in 2 years of follow‐up (MRI)	The *APOE* epsilon 4 allele were associated with higher hippocampal atrophy rates [178].
Saira Saeed Mirza, et al. (2011)	28 AD patients (*n* = 14; 50%) and 28 control subjects	Hippocampal atrophy (MRI)	*APOE*ɛ4 alleles aggravated brain atrophy [179].
S. S. Mirza, et al. (2019)	289 SDC patients (239 AD+ 50 DLB; *n* = 167, 57.79%)	Association of *APOE4* and white matter hyperintensities	Greater WMH volume was associated with worse attention/executive functions, learning/memory, and language in *APOEε4* carriers) [180].
X. Wang, et al. (2015)	42 AD patients (*n* = 16; 38.09%)	Hippocampal volume, and resting‐state functional connectivity in AD (MRI)	*APOE4* allele carriers exhibited smaller left hippocampal volumes compared to non‐carriers and decreased their amplitude of low‐frequency fluctuations in the left hippocampus [168].
U. Saeed, et al. (2018)	250 AD patients and 48 DLB patients	Hippocampal volume (MRI)	Hippocampal volumes were smaller with increasing *APOEε4* dosage [169].
M. M. Dunk and I. Driscoll (2022)	297 AD patients (*n* = 202, 68.01%), 539 Late MCIs (*n* = 287, 53.25%); 249 Early MCIs (*n* = 122, 43.15%); 404 controlss (*n* = 109, 26.98%).	Total cholesterol and APOE‐related risk for AD	Total cholesterol was higher in *APOE4*+ compared to *APOE3* and *APOE2*+ (*p*s < 0.04) carriers [181].
H. Barthel, et al. (2011)	81 AD patients (*n* = 32; 39.51%) and 69 non‐demented controls (*n* = 12; 17.39%)	The positive ratio of florbetaben (^18^F) PET finding.	*APOE ε4* was more common in participants with positive PET images compared with those with negative scans [182].
*EEG*			
M. Lehtovirta, et al. (1996)	58 AD patients (*n* = 287, 58.54%) and 18 control	Relationship of EEG and *APOE* polymorphism in AD	*APOE* ɛ4 allele carriers showed a tendency towards more pronounced EEG slowing in AD patients [99].
V. Jelic, et al. (1997)	41 AD patients (*n* = 24, 53.25%) and 34 control	The ratio of alpha and theta absolute power and EEG coherence in alpha frequency band	*APOE*ɛ4 does not influence EEG slowing, but may be associated with selective decreases in functional connectivity as assessed by EEG coherence [183].
C. Babiloni, et al. (2006)	89 MCI subjects (*n* = 32; 35.96%), 103 AD patients (*n* = 52; 50.49%)	Relationships between the *APOE*ɛ4 allele and EEG rhythmicity	Amplitude of alpha 1 and 2 sources in occipital, temporal, and limbic areas was lower in subjects carrying the ɛ4 allele than in ɛ4 non‐carriers, which was true for both MCI and AD [103].
N. V. Ponomareva, et al. (2008)	50 AD patients, and 95 their unaffected relatives and unrelated individuals.	Relationship of EEG alterations in non‐demented individuals and *APOE* genotype and risk of AD	Patients carrying the *APOE*ɛ4 allele the decrease in alpha power was higher than in ɛ4 non‐carriers [102].
L. Canuet, et al. (2012)	125 AD patients (*n* = 60; 48%) and 60 elderly controls (*n* = 12; 20%)	Spectral density for six frequency bands and resting‐state oscillations and functional connectivity	The decrease in interhemispheric alpha connectivity in frontal and parieto‐temporal regions was APOE‐4‐related [184].
V. Gutierrez‐de Pablo, et al. (2020)	46 healthy control subjects (*n* = 6; 13.04%), 39 MCI (*n* = 10;25.64%), 122 AD (n = 50; 40.98%)	Lempel‐Ziv complexity	*APOE*ɛ4 allele could modify the EEG complexity patterns in different brain regions, as the temporal lobes [101].
*Gait*			
R. K. MacAulay, et al. (2016)	299 non‐demented older adults (*n* = 75; 25.08%)	Gait characteristics	APOE‐e4 was linked to shorter stride length and greater dual‐task related disturbances in stride length [128].
H. E. Whitson, et al. (2018)	29 older adults with normal cognition	Gait‐cognition dual‐task performance	*APOE*ɛ4 carriers tended to exhibit greater dual‐task interference [185].
*Biomarkers* (*Aβ1‐42*; *p‐Tau*; *NFL*)			
T. Lehtimäki, et al. (1995)	83 AD patients and 164 non‐demented controls	*APOE* concentrations in the cerebrospinal fluid in Finnish patients with Alzheimer's disease	CSF *APOE* concentrations did not vary in different phenotype groups [186].
Y. Liu, et al. (2016)	336 AD patients (*n* = 223; 66.37%); 866 MCI patients (*n* = 436; 50.35%); 561 controls (*n* = 147; 28.49%)	Impact of ε4 dose on cerebrospinal fluid (CSF) levels’ Abeta1‐42 (Aβ1‐42), tau, p‐tau; cortical amyloid deposition (Florbetapir‐PETAV45)	*APOEε4* was associated with decreased CSF Aβ1‐42 and increased cerebral Aβ deposition, increased CSF tau, p‐tau and cerebral hypometabolism, hippocampal atrophy, and cognition decline [35].
M. Mandecka, et al. (2016)	85 SCD (*n* = 28; 32.94%), 87 MCI (*n* = 28; 32.18%), and 80 AD‐D (*n* = 43; 53.75%)	CSF biomarkers	The levels of T‐tau and P‐tau were significantly higher in the APOEɛ4+ than in the noncarriers, but only in the MCI patients (*p* < 0.05) [50].
J. K. Morris, et al. (2017)	213 non‐demented controls (*n* = 54; 25.35%), 125 AD patients (*n* = 78; 62.4%)	Metabolic biomarkers	APOE4 carriers with AD exhibited lower FFA levels [187],
*Neuroinflammation*			
Y. Y. Fan, et al. (2017)	185 AD patients, and 190 healthy individuals	TNF‐α, IL‐6, and IL‐1β.	The *APOE4* ε4 allele carriers have higher level of increased levels of TNF‐α, IL‐6, and IL‐1βin AD [86].
Qiushan Tao, et al. (2018)	3130	Study the interaction between the apolipoprotein E (ApoE) genotype and chronic low‐grade inflammation and its association with the incidence of AD.	*APOE4* coupled with chronic low‐grade inflammation was associated with an increased risk of AD [87].
John M Ringman, et al. 2012	33 FAD patients (*n* = 21; 63.63%)	Plasma inflammatory factors.	The *APOE* genotype was related to the levels of the inflammatory markers I‐309, IL‐1, IL‐3, IL‐7, IL‐12p40, IL‐13, and IL‐15 [82].

The regulation efficiency encoded by APOE isoforms executes diversely in CL synthesis and redistribution. Multiple microRNAs of astrocytes play a role in regulating CL synthesis in neurons by increasing histone acyl‐coenzymes and stimulating CL metabolism [28]. However, miRNA levels in *APOE4* carriers are much lower than those in *APOE3* carriers, which leads to higher CL levels in *APOE4* carriers [28]. Furthermore, compared to *APOE3*, the synthesis and secretion of CL decrease in *APOE4* overexpressed epithelial cells and increases in lysosomes; hence, CL produced by *APOE4* carriers is degraded more easily [29]. Additionally, poorly lipidated APOE is more likely to be decomposed [30]. As for the different domain interactions, APOE3 binds to small HDLs, whereas APOE4 prefers large VLDLs [31]. Consequently, APOE4 can degenerate more easily than APOE3, which negatively affects lipid metabolism in CNS and can eventually cause neural damage.

## 3 APOE AND AD PATHOLOGY

### 3.1 APOE and Aβ

Extracellular amyloid plaques are one of the pathological hallmarks of AD, which are formed by amyloid‐β (Aβ) accumulation, oligomerization, and deposition. Derived from the sequential proteolytic processing of the amyloid precursor protein (APP), Aβ contributes to neurotoxicity after deposition. An autopsy cohort study reported that relative to *APOE3* homozygotes, *APOE4* is associated with more Aβ plaques and cerebral amyloid angiopathy (CAA), while *APOE2* displayed lower Aβ plaques burden and CAA [32]. Consistent with this result, another research revealed that *ApoE4* carriers had the highest percentage of Aβ lesions at all ages, and *APOE4* carriers demonstrated Aβ deposits in their 40s [33]. APOE is associated with neuroinflammatory amyloid plaques [34, 35]. Unlike ApoE3, the basement membranes formed by APOE4 astrocytes favor the aggregation of Aβ [36]. APOE isoforms differentially mediate Aβ deposition, resulting in an isoform‐dependent effect on AD progression. The mechanism can be divided into APP synthesis and formation and clearance of Aβ.

APOE promotes amyloid plaque generation by increasing the synthesis of APP (rank APOE4>APOE3>APOE2) [37]. The primary mechanism is that the process of APOE binding to its receptors activates the signal of APP transcription. First, APOE binding to its receptors causes a 2–4‐fold increase in the level of dual leucine zipper kinase (DLK) [37]. DLK is highly expressed in neurons and plays a part in axon growth, apoptosis, and neuron degeneration [38]. Increased DLK levels lead to phosphorylation of MKK7 (a member of the MAPK signaling pathway); then, phosphorylated MKK7 motivates phosphorylation of extracellular signal‐regulated kinase (ERK1/2), ultimately stimulating transcription factor AP‐1 in the nucleus [37]. Importantly, AP‐1 can mediate the stimulation of APP transcription by APOE and promote a 2–6‐fold increase in c‐Fos phosphorylation, resulting in enhanced APP synthesis [37]. Supported by both in vivo and in vitro experiments, the same isoform‐specific differences (APOE4 > APOE3 > APOE2) were observed in each of the above processes [37], proving the significant role of APOE4 in AD pathogenesis.

In addition, these three isoforms function differently in the formation and clearance of Aβ. The inhibitory effect of APOE4 on Aβ peptide formation is worse than that of APOE3 [39, 40]. In addition, studies on mice demonstrated that APOE4 could decrease Aβ clearance compared to APOE3 [41, 42]. An in vitro trial also found that ApoE4 impaired autophagy in astrocyte cultures, and this effect was associated with a reduced capacity to clear Aβ plaques [43]. Furthermore, lipid‐free APOE3 and APOE4 can bind to Aβ and form stable complexes that obstruct the degeneration of Aβ so that Aβ binds more rapidly and effectively with APOE4 [44, 45]. Therefore, APOE contributes to AD in an isoform‐specific way.

### 3.2 APOE and the Tau protein

Microtubule‐associated proteins and tubulin comprise the microtubule system, which is an essential component of the neuronal cytoskeleton. Tau protein is tubulin with the highest content, which can promote protein assembly, stabilize and polymerize microtubules, and participate in neurite growth and axonal transport [46]. In brains suffering from AD, Tau is hyperphosphorylated and is associated with neuronal degeneration and loss [47], which are the main pathological features of AD [16]. The degree of Tau phosphorylation varies with APOE isoforms; for instance, *APOE4* knock‐in (KI) mice generated more Tau phosphorylation than *APOE3* KI mice [47, 48]. Furthermore, these isoform‐dependent differences vary with neuron type. In *APOE4* KI mice, Tau phosphorylation was increased in neurons, whereas there was no significant change in astrocytes [16, 49]. In addition, clinical researches also reported a higher level of T‐tau and P‐tau in APOE4 carriers in mild cognitive impairment (MCI) and prodromal AD stage patients [35, 50]. Although the mechanism of cell type‐specific phosphorylation is unclear, it provides strong evidence for differences in neural function among various APOE genotypes.

### 3.3 Lipid metabolism

**FIGURE 2:**
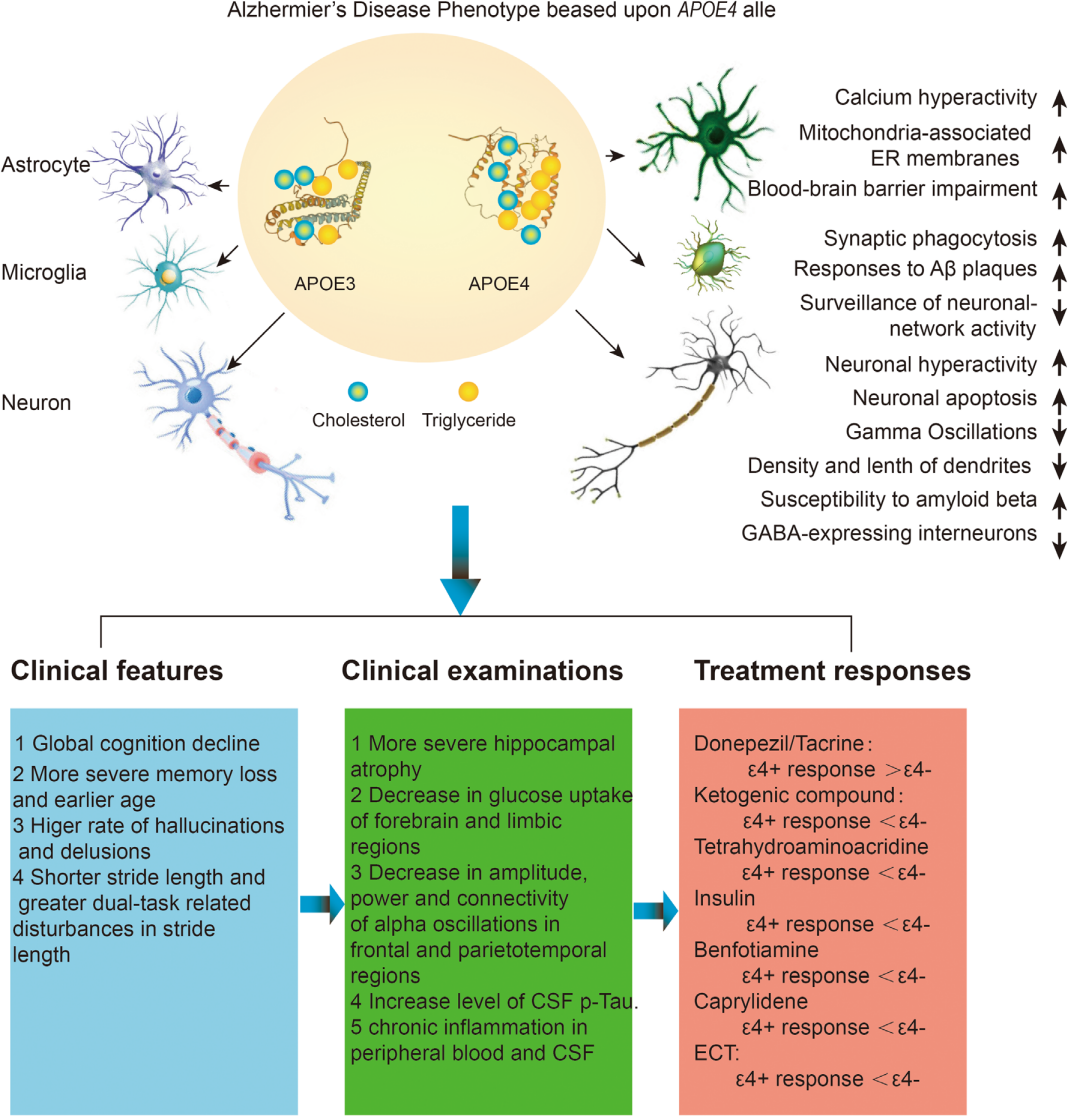
Genetic variation in the APOE genotype affects lipid metabolism and neurological development and producing relative isoform‐dependent changes of neurons, astrocytes, and microglia. These structural and neurophysiological changes form the basis of the clinical phenotype are defined by the carrier status of the apolipoprotein E ɛ4 allele.

Aging is a significant risk factor in AD, in addition to genetic and lifestyle factors [51]. Accumulated evidence indicates dysregulated lipid homeostasis that related to aging plays an important role in the development of AD [52]. Genetic variations in APOE genotype affected lipid metabolism and neurological development (Figure 2). Lipid and sterol synthesis and metabolism pathways are downregulated in *APOE4‐*carrying astrocytes but upregulated in those expressing APOE2 and APOE3 [29]. The total content of CL, cellular CL, and secreted CL were all decreased in *APOE4‐*carrying astrocytes (level E2 = E3 > E4 = KO) in APOE knock‐out (KO) mice [29]. Additionally, for proteins that play a vital role in lipid metabolism, western blot analysis has confirmed that Farnesyl‐Diphosphate Farnesyltransferase 1 (FDFT1) (squalene synthase) and ATP Binding Cassette Subfamily A Member 1 (ABCA1) are medicated in an APOE isoforms‐dependent way (E2 > E3 > E4) [29]. Cellular cholesterylester decreased significantly only in *APOE4*, whereas cellular triacylglycerol (TAG) increased in all APOE genotypes, and cellular phosphatidylethanolamines (PEs) increased only in *APOE2* and *APOE4* cells. Both exhibited an isoform‐dependent increase (E4 > E3 > E2 = KO) [29]. Moreover, *APOE4*‐expressing cells exhibit enhanced inflammatory signaling and decreased β‐amyloid uptake [29].

Genetic variations in *APOE* genotype lead to different fates in neurons. Lipid homeostasis can affect many cellular functions, including membrane synthesis, vesicle transport, protein transformation, and cell proliferation. *APOE4* KI mice revealed a decreased phagocytic capacity in astrocytes and increased senescent synapses compared with other isoforms in KI mice [53]. With decreasing CL secretion in astrocytes, the mice depicted fewer synaptic vesicles, more immature synapses, and less presynaptic synaptosomal nerve‐associated protein 25 (SNAP‐25) in the hippocampus [54]. In neurons, lipid metabolism is genotype dependent. Among the alleles, *APOE4* encodes the worst efficiency in lipid transport and lipid droplet accumulation [55]. In contrast, human *APOE2* and *APOE3* alleles can functionally replace the glial lazarillo (Glaz) loss in flies, thereby promoting lipid transport from neurons to glial cells [55]. The evidence indicates that APOE4‐carrying neurons are vulnerable to lipid metabolism disorders. Reduced binding affinity with lipids in APOE4‐carrying neurons also leads to lipid accumulation and subsequent hippocampal atrophy and cognitive deficits due to apoptotic neuronal death [56]. Pathological events also can aggravate the process in reverse. For example, elevated ROS can induce the lipids production in the neurons, which are subsequently transferred to glial cells and generate lipid droplets [55].

Variations in lipid and neuronal homeostasis critically affect neurological development and synaptic formation. High *APOE3* expression stimulates synaptic elongation, while *APOE4* sharply hampers synaptic branching and extension and impairs the process of cytoskeleton [57, 58]. Cerebral organoids from AD patients carrying APOE ε4/ε4 depicted greater apoptosis and decreased synaptic integrity [59]. Although it is still unclear how *APOE4* inhibits synaptic branching and extension, Ca^2+^ overload may be part of its mechanism. Ca^2+^ overload triggered by APOE result in Ca^2+^ concentration rising and CaMKII abnormal phosphorylation, and finally aggravate oxidative stress and damaged neurons apoptosis [60]. GABAergic neurons system mainly involves cognitive processes, especially learning and memory [61]. GABAergic neural network is also affected by *APOE* genotypes. Compared with *APOE3*, dysfunction in APOE4 carrying GABAergic interneurons result in hippocampal neurogenesis and deficits in learning and memory [62]. Clinical studies have found that GABA levels in the brain and cerebrospinal fluid (CSF) are diminished in patients with AD and are more serious in *APOE4* carriers [63, 64]. Meanwhile, *APOE4* genotype has been associated with increased brain activity at rest and responses to memory tasks, proving the impairment of GABAergic neurons [65, 66]. In addition, *APOE* genotype attaches great significance to mature hippocampal neurogenesis. GABAergic neurons are reduced in *APOE4‐*carrying brains, resulting in decreased GABA input to newborn neurons and inhibiting neurogenesis and maturation of neural stem cells [67]. Moreover, compared with *APOE3*, *APOE4*‐expressing GABAergic neurons displayed a reduction in their growth, number, and branching of dendrites [68]. However, the specificity of *APOE4* isoforms varies according to the susceptibility of neurons toward stress conditions and the effects of *APOE4* in different brain regions [68, 69]. In brief, these findings demonstrate that *APOE4* may cause age‐dependent damage to GABAergic interneurons, resulting in reduced hippocampal neurogenesis, as well as learning and memory deficits.

Last but not least, myelination, differentiation of oligodendrocytes, in CNS neuronal axons is linked to CL disorder resulted by APOE4. Through differential expression and gene set enrichment analysis of the postmortem samples, it is evidenced that expression of cholesterol‐related genes in oligodendrocytes is raised in a APOE4 dose dependent way [70]. Another study implies that myelination might be affected by altered levels of intracellular and extracellular cholesterol of oligodendrocyte [71]. The mechanism how APOE4 affect myelination can be explained in two approaches. First, APOE4 directly interferes oligodendrocytes by changing its lipid composition, increasing lipid droplet synthesis and impairing cholesterol trafficking and subcellular localization [70]. It is found that cholesterol is abnormally deposition in myelinating oligodendrocytes [72]. Secondly, studies suggested that APOE4 indirectly disrupts oligodendrocyte differentiation by means of influencing astrocyte‐derived lipid transport [73]. However, the pathway is needed for further exploration.

### 3.4 Blood‐brain barrier (BBB)

Blood‐brain barrier is mainly composed of brain capillary endothelial cells (BMEC), pericytes, astrocytes, perivascular foot and basement membrane. The integrity of BBB can limit the free diffusion of ions between blood and brain tissue, preventing harmful substances from entering brain tissue. Recent studies found that the permeability of BBB increased by 40% in the brain of AD patients, possibly related to the injury and even death of vascular endothelial cells and pericytes. Moreover, damage to vascular endothelial cells and pericytes is closely related to the occurrence of dementia. *APOE4* can lead to an increase in amyloid protein at the peripheral level, which is related to the destruction of the BBB to a certain extent. In *APOE* knock‐in and glial fibrillary acidic protein promoter transgenic mice, it was found that *APOE4* activated the proinflammatory cytokines cyclophilin A in pericytes and NF‐KB/matrix metalloproteinase‐9 signaling pathways, thereby increasing susceptibility to BBB impairment [74].

### 3.5 Neuroinflammation

Neuroinflammation is major pathogenesis of AD [75]. Multiple immune cells, including microglia, are involved in the process, and APOE could exacerbate the neuroinflammatory process [76, 77]. APOE deficiency in mice is associated with Aβ‐related inflammatory responses [78], and APOE isoforms may modulate inflammatory responses differently [79]. For example, *APOE4* knock‐in mice were more susceptible to inflammation caused by lipopolysaccharides or Aβ deposition than *APOE2* and *APOE3* knock‐in mice [80, 81, 82]. *APOE4* mice are also more susceptible to brain injury with a strong inflammatory component, such as traumatic brain injury [83]. Similar results have also been obtained from *in vitro* trials: APOE affects the inflammatory process of microglia and astrocytes, while *APOE4* has the strongest pro‐inflammatory effect [84, 85]. Besides, it was also found that APOE4 ε4 allele may enhance susceptibility to AD and promotes the expressions of inflammatory factors in AD patients [86], and ApoE4 coupled with chronic low‐grade inflammation was associated with an increased risk of AD [87]. A previous study reported that AD patients with APOE4 allele exhibited increased activation of the eicosanoid lipidome during chronic inflammation, which was identified as a potential therapeutic target for resolving this chronic inflammatory state [88].

In summary, the differences in inflammatory cytokines are related to the different APOE phenotypes, not only in CNS but also in the peripheral blood. However, the specific mechanism by which APOE genotypes regulate inflammatory response remains unclear and should be investigated.

## 4 CLINICAL FEATURES BASED ON APOE4 STATUS

### 4.1 Gender difference

As a basic feature, gender is a significant factor in analyzing a disease. Statistically, in China, the prevalence and mortality of AD are remarkably higher in females than males [89]. Many contributors to this sex difference have been studied, such as education, occupation, menopause and so forth [90]. It is reported that women carrying *APOE4* have a greater risk than men with the same APOE genotype [91]. Nevertheless, the nature and direction of *APOE4* related to gender discrepancies remain controversial. Comprehensive research suggested that women carrying *APOE4* may show greater levels of AD pathology [92], more serious brain network integrity [93] and faster cognitive decline [94]. Furthermore, functional brain connectivity in healthy elders found that female *APOE4* carriers demonstrated reduced functional connectivity compared with male *APOE4* carriers in a cuneus/precuneus cluster of the posterior default mode network [95]. However, some studies reported the opposite results. A recent study implied that carrying *APOE4* influences cognitive decline to the same degree in two genders, while the dose‐dependent effects of *APOE4* on cognitive decline, and the worsening of these effects with age, are stronger in men than women [96]. Otherwise, no difference between sexes carrying *APOE4* is also suggested [95, 96]. Overall, the interaction between gender and the number of *APOE4* may be complicated and should consider other factors (age and vascular risk factors) while analyzing.

### 4.2 EEG alterations (neural network)

Electroencephalography (EEG) metrics are a critical early biomarker of preclinical AD [97]. Specifically, patients suffering from AD have decreased α coherence in temporal, occipital regions and parietal and increased δ coherence in the frontal and parieto‐occipital regions [98]. As displayed in Table 1, recent EEG studies have found that APOE genotype can affect the neural network. Lehtovirta et al. found that *APOE4* carriers had more pronounced EEG slowing than non‐carriers in patients with early AD [99]. *APOE4* homozygotes demonstrated the lowest fast‐wave amplitudes, and highest slow‐wave value in relative amplitudes and the lowest mean and peak frequencies after three years of follow‐up [100]. Similarly, Ponomareva et al. suggested that *APOE4* carriers significantly reduced α power more than non‐carriers in patients with AD. In addition, in the case of hyperventilation, the presence of the ε4 allele in relatives of patients with AD is associated with synchronized high‐amplitude δ and θ activity and sharp wave performance, with a decrease in α and an increase in δ and θ relative power [101, 104]. In other words, *APOE4* allele may increase the abnormal EEG rate in AD patients and their relatives without cognitive dysfunction.

There was also evidence of a significant difference between APOE4 carriers and non‐carriers, where an increase in a θ‐α band in the left temporal region could be seen in *APOE4* carriers [102]. Meanwhile, in patients with MCI and AD, the amplitude range of α1 and α2 in the occipital, temporal and limbic regions of *APOE4* carriers was lower than that in non‐carriers [103]. Overall, the presence of *APOE4* allele will likely increase excitability and accelerate dysfunction. These changes occurred before the first clinical symptoms. Consequently, APOE genotype may be a neurophysiological endophenotype.

### 4.3 MRI (brain structure)

AD progression is characterized by significant atrophy (or cortical thinning), mostly in AD‐susceptible areas such as the medial temporal lobe [104]. Senile plaques and neuronal tangles can appear in the medial temporal lobe (including the hippocampus and entorhinal cortex) in the early stages of AD. An entorhinal cortex‐hippocampus projection fiber may be involved in hippocampal atrophy [105]. According to an MRI study, *APOE4* allele was associated with greater hippocampal atrophy; the degree of atrophy was higher in *APOE4* carriers than in non‐carriers, especially in the medial temporal structures [106]. Moreover, APOE2 carriers had larger cortical thickness than APOE3 carriers in the temporal cortex, as well as larger cortical thickness than APOE4 carriers in the dorsolateral prefrontal cortex [107, 108]. However, these findings are inconsistent with other studies. The Alzheimer's Disease Neuroimaging Initiative (ADNI) study of subjects with AD and MCI did not find any significant effect of *APOE4* on atrophy [109]. Another study with a small number of AD patients found that APOE4 significantly affected the dentate gyrus and CA3, but these areas were considered less affected by AD [110]. APOE4 may contribute to increased hippocampal atrophy; however, this association is unclear (Table 1).

### 4.4 FDG‐PET metabolic patterns

The ^18‐^fluorodeoxyglucose PET (FDG‐PET) imaging method measures the cerebral metabolic rates of glucose (CMRglc), a critical index for neuronal activity that correlates with disease progression and predicts histopathological diagnosis [111]. Few studies are exploring the effect of *APOE4* on FDG‐PET. However, the overall conclusions are similar: *APOE4* carriers demonstrated a greater decrease in brain metabolism than non‐carriers [112]. In addition, a metabolic decline has been observed in regions sensitive to AD (mainly the posterior cingulate, parietal, and temporal lobes) but was also found in the prefrontal cortex [113]. Similar results were observed in young (20–39 years) *APOE4* carriers [114]. Most importantly, the *APOE4* allele was found to have a gene‐dose effect on brain metabolism, in which *APOE4* homozygous individuals exhibited more decreased brain metabolism than heterozygous ones [115]. Although the available data suggest that *APOE4* is associated with decreased metabolism in AD‐sensitive brain regions compared to APOE2 and APOE3, this feature requires further investigation.

### 4.5 Neuropsychiatric symptoms

Neuropsychiatric symptoms are an important clinical feature of AD and cognitive impairment. Patients can suffer from psychosis (i.e., delusions and hallucinations) as well as affective and behavioral changes (i.e., depressive mood, anxiety, irritability, apathy, euphoria, disinhibition, and agitation) [116]. Depression and anxiety are common, even in the early stages of AD or MCI [117]. From a cohort of 112 patients with Alzheimer's dementia evaluated by the Neuropsychiatric Inventory (Nursing Home Version, NPI‐NH), 92.9% had at least one neuropsychiatric symptom [118]. However, the association between APOE genotype and NPS in AD, whether *APOE4* increases anxiety and depression in AD, remains controversial. The prevalence rate of depression in *APOE4* allele carriers is significantly higher than in non‐carriers, especially in female *APOE4* allele carriers [119, 120]. Nevertheless, this is not always the case. *APOE4* allele has depicted a protective effect on depression [121]. Studies with large sample sizes may be needed to investigate the differences in neural networks and the relationship between APOE genotypes and neuropsychic behavior abnormalities.

### 4.6 Gait

Gait is associated with cognitive function in the elderly, especially AD patients. Cognitive impairment related to frontal lobe cognition in AD may lead to disturbances in the gait and motor parameters [122, 123, 124]. Kinematic parameters of gait are associated with an increased risk of falls in patients with AD. Compared with normal elders, people with AD are three times more at risk of falling [125], fractures, reduced mobility, and loss of independence, leading to increased cardiovascular morbidity and mortality [126, 127].

There are few studies on the correlation between gait and *APOE4*. One study found that *APOE4* carriers had shorter step sizes and greater dual‐task‐related step size interference [128]. APOE genotypes may also affect men and women differently through their effects on early disease processes, such as hypercholesterolemia, and these diseases may subsequently have a potential impact on AD pathogenesis [128]. Other longitudinal studies have demonstrated that decreased motor function in older adults predicts subsequent cognitive decline, and these changes are related to a greater genetic risk for AD [128]. In conclusion, the *APOE4* allele is likely to affect the gait characteristics of AD. This relationship has not been clarified, which requires thorough exploration.

## 5 PRECISION MEDICINE BASED ON APOE4 CARRIER STATUS

According to the Precision Medicine Initiative, precision medicine is "an emerging approach for disease treatment and prevention that considers individual variability in genes, environment, and lifestyle for each person." *APOE4* is a major risk factor for AD, and strategies based on *APOE4* might hold promise within the precision medicine framework.

### 5.1 Diagnoses and prevention based on APOE genotype

Prospective biomarkers comprising Aβ42, t‐tau, p‐tau, tau/Aβ42 in CSF, as well as t‐tau, Aβ42/Aβ40, and NFL in peripheral blood, are related to AD progression, as summarized by a systematic review and analysis [129]. Otherwise, whole, left, and right HV, EC volume, MTA, 18 F‐FDG PET and 11 C‐PIB PET are prospective neuroimaging strategies applied in AD diagnoses [129]. According to the analysis, *APOE4* carrier has a great predictive ability for the progression with a RR of 2.16 and 95% CI of 1.83–2.55 [129]. Whether APOE genotype affects the accuracy of CSF biomarkers are evaluated by some research. A study reported that CSF levels of Aβ42 but not total and phosphorylated tau were lower in *APOE4* carriers than with noncarriers in AD and MCI patients [130]. However, CSF Aβ42 was strongly associated with diagnosing AD and cortical Aβ accumulation independent of APOE genotype [130].

On the other hand, *APOE4* demonstrated great potential in assessing the risk of cognitive decline and AD. In cognitively healthy older adults, *APOE4* can accelerate their age‐related memory decline with *APOE4* carriers earlier than ten years than non‐carriers in Aβ‐positive elderly [131] and progress earlier to MCI or AD [132]. Accordingly, *APOE4* might help evaluate the potential risk of age‐related cognitive decline and AD, especially in Aβ‐positive people. When Aβ tests positive, APOE gene tests are strongly suggested and take interventions based on the gene test result.

In summary, the sensitivity and specificity APOE gene test are relatively low in diagnosing AD, while APOE4 has moderate diagnostic value and promising applications in preventing AD [133].

### 5.2 Responses to clinical treatment based on APOE4 status

The differences in the structure and function of APOE genotypes determine differences in the evolutionary processes and fates of the neural network, metabolism, and other aspects of the nervous system. This can also determine the different responses to multiple treatments (Table 2). *APOE4* carriers respond differently to treatment than non‐carriers. For example, nonsteroidal anti‐inflammatory drugs can lower the risk of AD in *APOE4* carriers but not in non‐carriers [134]. Two‐phase III trials of Bapineuzumab in AD displayed the same result: although Bapineuzumab did not change the overall clinical outcome in patients with AD, improvements in markers related to hyperphosphorylated Tau and amyloid plaque deposition were observed in *APOE4* carriers [135]. Another study revealed the sensitivity of *APOE4* non‐carriers to drug treatment. Amyloid Aβ was significantly reduced in *APOE4* non‐carriers but not in carriers after treatment with the retinoid X receptor (RXR) agonist bexarotene [136]. Moreover, a lasted phase IIa clinical trial of benfotiamine reported a stronger efficiency in APOE4 non‐carriers than carriers [137]. In addition, phase II clinical trials of intranasal insulin in AD and MCI also reported different treatment effects modulated by APOE genotype status [138, 139]. Although different drug treatment mechanisms do not remain the same, carriers and non‐carriers exhibit different drug treatment responses. Regarding non‐drug therapy, Naili Wei et al. discovered that non‐carriers of the *APOE4* allele were more sensitive to transcranial magnetic stimulation treatment in an AD RCT project, which can probably be explained as the difference in the neural network due to different genotypes [140]. Another study found that physical exercise was strongly associated with reduced Pittsburgh compound B (PiB) positivity rates in cognitively normal *APOE4* carriers, suggesting that a sedentary lifestyle in *APOE4* carriers may increase the risk of amyloid deposition [141]. However, it was also reported conversely results. A study using walking and lower limb strength training as an intervention suggested no significant difference between APOE4 alle carriers and non‐carriers [142].

**TABLE 2:** Responses to therapeutics or interventions based upon APOE geneotype.

Authors (publish years)	Nubmers of patients included in studies	Treatment protocols	Main findings (*p* value)
*Drug therapy*			
S. Abushakra, et al. (2017)	2,025 AD patients (*n* = 112; 71.3%)	Randomized subjects received oral placebo, 100 mg BID, or 150 mg BID of tramiprosate.	The Mild subgroup of APOE4/4 AD patients showed larger benefits on the high dose of tramiprosate than the overall Mild and Moderate group (*p* < 0.02) [188].
S. H. Choi, et al. (2008)	51	5–10 mg of donepezil per day for 48 weeks	APOE ε4+ may respond more favorably to donepezil thanε4 noncarriers (*p* ≤ 0.05) [189].
M. R. Farlow, et al. (1999)	959	once‐daily placebo (*n* = 374) or metrifonate (30–60 mg based on weight or a 50‐mg fixed dose, *n* = 585)	APOE genotype did not influence disease progression as evaluated by either cognitive performance (*p* = 0.93) or global function (*p* = 0.64) [190].
M. R. Farlow, et al. (1996)	460 (APOEε4‐carriers:291; noncarriers:169)	Placebo or tacrine at dosages of 80, 120, or 160 mg/day	APOEε4 associated with a lower probability of cognitive improvement (*p* ≤ 0.05) [191].
M. R. Farlow, et al. (1998)	528	Placebo or tacrine with daily dosages of 80, 120, or 160 mg/day	The treatment effect only found larger in the ε2‐3 compared with ε4 women (ITT, 4.24 points, *p* = 0.03; evaluable, 7.20 points, *p* = 0.01) [192].
M. Gold, et al. (2010)	693	Once‐daily placebo, 2 mg rosiglitazone extended release (RSG XR), 8 mg RSG XR or 10 mg donepezil (control)	No evidence of efficacy of 2 or 8 mg RSG XR monotherapy in cognition or global function was detected in the APOEε4− or other analysis populations [193].
S. T. Henderson, et al. (2009)	152	An oral ketogenic compound, AC‐1202	Effects were most notable in APOEε4− who were dosage compliant (*p* < 0.05) [194].
M. A. Raskind, et al. (2000)	636	Placebo or galantamine 24 or 32 mg/day	Therapeutic response to galantamine was not affected by APOE genotype [195].
M. A. Reger, et al. (2006)	13 AD, 13 with amnestic mild cognitive impairment, 35 normal controls	Saline (placebo) or insulin (20 or 40 IU)	For memory‐impaired subjects: APOEε4− > APOEε4+ (*p* < 0.05) [196].
A. S. Rigaud, et al. (2000)	76 (33ε4+; 43ε4−)	Tacrine dosages ranging from 40 mg/day up to the highest dosage (160 mg)	There was no tendency for the ε4− carriers to respond better than the ε4+ carriers [197].
M. E. Risner, et al. (2006)	511	Placebo or rosiglitazone (RSG) 2, 4, or 8 mg	Improvement in response to RSG: APOE ε4− >APOE ε4+ (only in exploratory analyses) [198].
G. H. Suh, et al. (2006)	202	Galantamine	ApoE epsilon4 genotype does not affect galantamine‐related improvements in cognition, global rating, function and behavior [199].
Q. Xu, et al. (2020)	53	Medium‐chain triglycerides (MCT) jelly or placebo jelly (canola oil) by mouth three times daily	MCT had positive effects on cognitive ability in mild to moderate AD patients with APOE4(−/−) (*p* < 0.05) [200].
O. Almkvist, et al. (2001)	24	Tacrine, a cholinesterase (ChE) inhibitors and placebo	The frequency of APOEε4 alleles was higher in responders (*p* < 0.05) [201].
X. A. Alvarez, et al. (1999)	30 patients with mild to moderate senile dementia	Citicoline or placebo	The efficacy of citicoline is greater in patients with mild mental deterioration and/or bearing the APOEε4 (*p* < 0.05) [202].
T. Babić, et al. (2004)	84	Galanthamine, a new cholinesterase inhibitor	APOE4 homozygous patients with AD in its mild to moderate stage may be considered as responders to galanthamine (*p* = 0.032) [203].
R. Blesa, et al. (2006)	214	Rivastigmine 1.5–6 mg twice daily for 26 weeks	APOE ε4 allele does not determine a difference in the response to treatment with rivastigmine [204].
L. De Beaumont, et al. (2016)	Tissues from temporal cortex (*n* = 37) and hippocampus (*n* = 22) from AD‐confirmed cases	Donepezil or placebo	APOE‐ɛ4 (*p* = 0.07) and butyrylcholinesterase K (KBCHE‐K) (*p* = 0.036) positive subjects show a greater benefits to donepezil therapy [205].
S. I. Gavrilova, et al. (2005)		Neurotrophic (cerebrolysin) and cholinergic (exelon)	APOEε4+ did not differ in response to either drug from APOEε4− [206].
H. J. Han, et al. (2012)	206	Rivastigmine patch monotherapy or memantine plus rivastigmine patch for 24 weeks	Moderately severe AD patients with the APOE ε4 allele may respond more favorably to memantine plus rivastigmine patch than ε4 noncarriers (*p* < 0.001) [207].
C. Harrington, et al. (2011)	2981	Once daily placebo, 2 mg rosiglitazone extended release (RSG XR), or 8 mg RSG XR for 48 weeks	There was no evidence of an interaction between treatment and APOE status [208].
A. S. Rigaud, et al. (2002)	117	Donepezil	No evidence show that APOE phenotype and gender are predictors of the response to donepezil in Alzheimer's disease patients [209].
C. R. Jack, et al. (2008)	131	Vitamin E and donepezil	APOEε4+ show greater annual percent change (APC) than APOEε4− (*p* < 0.000) [210].
Y. Zhong, et al. (2013)	110	5–10 mg of donepezil daily for 6 months	No association was found between the APOE genotype and efficacy of donepezil [211].
J. Jia, et al. (2020)	241	Donepezil 5 mg/day for at least 4 weeks	Patients' MMSE scores improved significantly after treatment (*p* = 0.0038), especially for APOEε4− and patients ≤75 years [212].
L. S. Schneider and M. Farlow (1997)	318 (all female)	Placebo or tacrine	Among women on estrogen replacement therapy (ERT) receiving tacrine, there tended to be greater improvement relative to placebo among those without an APOEε4 allele [213].
S. H. MacGowan, et al. (1998)	107	Tacrine or galanthamine	APOE genotype did not modify response to therapy in the short term, there are indications that it may affect response over the longer term (up to 12 months) (*p* < 0.05) [214].
M. Sjögren, et al. (2001)	145	Tacrine	It showed a faster rate of decline in the ApoE4 AD compared to the ApoE2‐3 (*p* < 0.05) [215].
E. Stefanova, et al. (2003)	27	Rivastigmine or tacrine	The CSF‐tau changes were mainly seen in APOEε4 carriers (*p* = 0.005) [216].
N. Pomara, L. M. Willoughby, K. Wesnes and J. J. Sidtis (2004)	24	Trihexyphenidyl or placebo	APOEε4 allele plays a significant role in increasing cognitive sensitivity to trihexyphenidyl (*p* = 0.01) [217].
P. Riekkinen, et al. (1997)	19	Tetrahydroaminoacridine (THA)	APOE genotype may affect the response of cortical electrical arousal to cholinergic therapy that enhances the efficacy of presynaptic NB axons (APOEε4− > APOEε4+, *p* < 0.05) [218].
S. Y. Tai, et al. (2017)	60	Cilostazol	APOE ε4 status, were significantly associated with poor therapeutic outcomes [219].
G. S. Watson, et al. (2009)	16	Insulin (1 mU.kg(−1).min(−1)) and dextrose to maintain euglycemia; octreotide (150 μg/h); insulin, dextrose, and octreotide; saline.	APOEε4 genotype modulates responses to insulin and octreotide (*p* < 0.05) [220].
G. E. Gibson, et al. (2020)	70	Benfotiamine or placebo	The efficiency of benfotiamine was stronger in the APOEɛ4 non‐carriers (*p* < 0.0001) [137].
I. C. Arellanes, et al. (2020)	33	Vitamin B complex (1 mg vitamin B12, 100 mg of vitamin B6 and 800 mcg of folic acid per day) and randomized to 2152 mg of docosahexaenoic acid (DHA) per day or placebo over 6 months	The increase in CSF eicosapentaenoic acid (EPA) in non‐APOE4 carriers after the DHA supplementation was three times greater than APOE4 carriers (*p* < 0.05) [221].
A. S. Fleisher, et al. (2011)	313	Divalproex or placebo	No baseline differences were found between active treatment and placebo groups in APOE ε4 carrier status [222].
A. Valen‐Sendstad, et al. (2010)	55	1‐mg estradiol and 0.5‐mg norethisterone or placebo once daily	Women without an APOEε4 allele may get better mood and cognition with hormone therapy (HT) (*p* < 0.05) [223].
R. Cacabelos, et al. (2000)	479	CDP‐choline (1000 mg/day) + piracetam (2,400 mg/day) + anapsos (360 mg/day)	APOE‐3/4 carriers showed the best efficiency, while APOE homozygote the reacted the worst (*p* < 0.05) [224].
S. Craft, S. Asthana, et al. (2003)	37	Insulin	AD patients who are not epsilon 4 homozygotes have reduced sensitivity to insulin that may interfere with such modulation (*p* < 0.05) [225].
A. Claxton, et al. (2015)	60	Placebo or 20 IU of insulin detemir or 40 IU of insulin detemir	This effect was moderated by APOE status (*p* < 0.05), reflecting improvement for APOE‐ε4 carriers (*p* < 0.02), and worsening for non‐carriers (*p* < 0.02) [226].
N. Torosyan, et al. (2018)	16	Caprylidene or placebo	APOEε4 non‐carriers showed greater improvement with caprylidene (*p* = 0.04) [227].
*Neuromodulation*			
A. Jannati, et al. (2017)	18 healthy adults	Continuous theta‐burst stimulation (cTBS)	No significant effect of APOE genotype. (FDR‐adjusted *p* > 0.32) [228].
M. Huuhka, et al. (2005)	119	Electroconvulsive therapy (ECT)	APOEε4+ and APOEε4− had no difference in response to ECT [229].
C. A. Bousman, et al. (2015)	117	Electroconvulsive therapy (ECT)	No association was found between APOE genotype and ECT efficiency [230].
*Other interventions*			
A. Solomon, et al. (2018)	1175	Diet, exercise, cognitive training, and vascular risk management	The APOE ε4 carriers and noncarriers were not significantly different at baseline (except for serum cholesterol level) [231].
L. M. J. Sanders, et al. (2020)	91 patients with dementia	Walking and lower limb strength training	No significant difference between APOEε4+ and APOEε4− .[142].
I. L. Uijen, et al. (2020)	67	Cycling training or stretching and toning exercises	There was no significant associations between APOE ε4 status and global cognitive change [232].

In conclusion, APOE4 carriers and non‐carriers respond differently to medical interventions, indicating that APOE genotype can be a guide of precision medicine.

### 5.3 Therapeutic strategies targeted on *APOE4* (and pathophysiology)

Currently, most studies on AD treatment methods target Aβ, but the results are unsatisfactory [123]. Therefore, new therapeutic targets and research directions are urgently required. APOE4 plays a vital role in AD pathogenesis, so APOE genotypes may be used as standards in clinical trials. A few studies have explored therapeutic targets based on APOE genotypes. A few studies have expored therapeutic approaches targets on APOE4, including immunotherapy, mimetic peptides therapy, structural correctors, gene therapies.

Immunotherapy is a promising way to decrease APOE4 and consequently alleviate Aβ plaque. HJ6.3 and HAE‐4 are two antibodies that have been previously studied. HJ6.3 is a monoclonal antibody specific against APOE [143]. Amyloid model mice demonstrated decreased Aβ levels and amyloid plaques after being administrated HJ6.3 [143, 144]. Anti‐human APOE4 antibody (HAE‐4) is an anti‐human antibody that specifically recognizes human APOE4 and APOE3 and preferentially binds nonlipidated, aggregated APOE over the lipidated APOE found in circulation. Administration of HAE‐4 in mice reduced Aβ plaques, Aβ‐driven tau seeding/spreading, and neuritic dystrophy [145, 146] while simultaneously protecting cerebrovascular integrity and function [147]. And mimetic peptide therapy is a strategy based on APOE structure and its biochemical interaction. Mimetic peptides are short peptide sequences that can compete for APOE binding, inhibiting APOE receptor binding and reducing its function [148]. Treatment with mimetic peptides significantly improved behavior while decreasing the inflammatory cytokine IL‐6, neurofibrillary tangle‐like and amyloid plaque‐like structures in transgenic mice [149, 150]. In addition, using small molecules as a structure corrector to disturb the interdomain interaction of APOE4 is also seen as a great therapeutic way. CB9032258 (a phthalazinone derivative) inhibits domain interaction in neuronal cells, which could restore functional activities of apoE4‐expressing cells [151]. Another study also found that a small‐molecule structure corrector could ameliorate the detrimental effects in APOE4‐expressing neurons [152]. Importantly, it is well known that *APOE4* is a risk gene, whereas *APOE2* is a protective gene, and *APOE3* is relatively normal. Hence, converting *APOE4* to *APOE2* or *APOE3* is a possible method for AD therapy. Induced pluripotent stem cells (iPSCs) study found that utilizing CRISPR/Cas9 (a genome‐editing system) to convert APOE4 to APOE3 was sufficient to attenuate multiple AD‐related pathologies [153]. However, CRISPR/Cas9 system still immaturity and needs more exploration.

In conclusion, although APOE4‐targeted therapeutic strategies still require further investigation, APOE4 targets could be considered promising therapeutic pathways for AD.

## 6 APOE PHENOTYPIC CLASSIFICATION

Phenomics mainly studies how physical and chemical phenotypes of organisms change under mutations and environmental influences to systematically investigate all cell phenotypes of genotypes in different environments [154]. It can effectively trace the associations between genotypes, environmental factors, and phenotypes [154]. Phenome‐wide association studies (PheWASs) are adopted to investigate one or more phenotypes associated with genetic variation [155]. Such phenomics research can help to discover risk and even pathogenic genes, determine different characteristics of diseases, facilitate drug application as well as achieve breakthroughs in precision medicine [155, 156].

AD treatment has long been unsatisfactory, and the failures make us constantly reconsider whether the original directions are correct. For instance, the failure of Aβ‐targeted therapies has encouraged people to view Aβ as a pathological condition rather than a mechanism. Meanwhile, there have been no significant breakthroughs in developing drugs treating Tau's hyperphosphorylation. Currently, treatments or various drugs for AD are based on a certain phenomenon or evidence without comprehensive and sufficient consideration [157]. Drug development has included a variety of complex and unclear mechanisms. However, we reflected on the results of clinical drug treatment and found that APOE genotype greatly influenced treatment responses (Table 2). For example, *APOE4* carriers have different sensitivity to various drugs [158]. The difference in APOE genotype on treatment led us to consider whether *APOE4* carriers and non‐carriers of *APOE4* should be classified into different phenotypic groups in clinical studies.

The phenotype group concept was proposed based on the different phenotypic characteristics of certain genes under the joint action of various epigenetic factors. The mechanism is the different regulation and modification of DNA expression by various epigenetic factors, including DNA and RNA methylation. According to the characteristics mentioned above, APOE genotypes appear to satisfy this characteristic. Specifically, APOE gene polymorphism leads to protein binding efficiency. In addition, different APOE phenotypes influence the epigenetic modification status, such as DNA methylation [159, 160]. As APOE is involved in synapses, ribosomes, mitochondria, spliceosomes, endocytosis, oxidative phosphorylation, and proteasome functions, the status/change of the two sites could greatly impact individuals [160]. Therefore, it is different in CNS and cardiac circulation system disease states among APOE carriers [161, 162], which is based on the environment or different stimulus conditions. Considering the above evidence, phenomics based on APOE genotypes is promising.

## 7 CONCLUSION

APOE genotypes encode the metabolic efficacy lipoprotein in astrocytes, neurons, and microglia. *APOE4* has a high binding affinity with triglycerides and CL, which differ from the ability of APOE3. Such variations produce extensive differences between *APOE4* carriers and non‐carriers in the neural network, pathological state, clinical features, imaging, electrophysiology, and treatment responses. All of these contribute to the two kinds of phenotypic features. Therefore, differentiating phenotypes based on *APOE4* carrier status should be considered. In future clinical studies, phenotypic classification should be applied to research and clinical treatments. These phenotypes will steer the direction of AD research to be more targeted and precise. Most importantly, this may pave the way for developing effective drugs.

## AUTHOR CONTRIBUTIONS

**Xiao‐Yu Ji, Xin‐Yuan Peng and Hai‐Liang Tang:** Writing and editing the manuscript. **Jie Wu and Jian Chen:** Language editing and major supervision. **Nai‐Li‐Wei:** Conception, supervision, and design of this article. All authors approved the submitted version.

## CONFLICT OF INTEREST STATEMENT

The authors declare no competing interests.

## Data Availability

The article is a review. It do not include any data.
